# Advances in liquid biopsy for bone and soft-tissue sarcomas

**DOI:** 10.1007/s10147-025-02813-2

**Published:** 2025-07-18

**Authors:** Yilang Wang, Tomohiro Fujiwara, Takanao Kurozumi, Teruhiko Ando, Takahiko Ishimaru, Hiroya Kondo, Eiji Nakata, Toshiyuki Kunisada, Toshifumi Ozaki

**Affiliations:** Department of Orthopaedic Surgery, Okayama University Graduate School of Medicine, Dentistry, and Pharmaceutical Sciences, Okayama, Japan

**Keywords:** Liquid biopsy, Bone sarcoma, Soft-tissue sarcoma, Circulating tumor cells, Circulating nucleic acids, Circulating microvesicles

## Abstract

Bone and soft-tissue sarcomas are a heterogeneous group of malignant tumors originating from mesenchymal tissues, accounting for approximately 1% of adult solid malignancies and 20% of pediatric solid malignancies. While blood-based tumor markers are available in major types of cancers, evidence demonstrating useful circulating biomarkers is limited in bone and soft-tissue sarcomas. Despite the development of combined modality treatments, a significant proportion of sarcoma patients respond poorly to chemotherapy or radiotherapy, leading to local relapse or distant metastasis. However, imaging methods, such as X-ray, computed tomography, positron emission tomography, magnetic resonance imaging, and scintigraphy, are mostly used to detect or monitor tumor development. Liquid biopsy is an emerging minimally invasive diagnostic technique that detects tumor-derived molecules in body fluids, including circulating tumor cells, circulating tumor DNA (ctDNA), circulating tumor RNA (ctRNA), and circulating extracellular vesicles. This method offers new possibilities for early tumor detection, prognostic evaluation, and therapeutic monitoring and may serve as a benchmark for treatment modification. This review focuses on the current technological advances in liquid biopsy for bone and soft-tissue sarcoma and explores its potential role in guiding personalized treatments. If these modalities could determine resistance to ongoing therapy or the presence of minimal residual disease at the end of the treatment protocol, the obtained data would be important for determining whether to change treatment approaches or add adjuvant therapies.

## Introduction

### Bone and soft-tissue sarcoma

Bone and soft-tissue sarcomas are a large class of primary malignant tumors originating from mesenchymal tissue, including bones and connective tissue throughout the body. Sarcomas account for approximately 1% of adult tumors and 20% of pediatric malignancies [1]. Nearly 15,000 cases in the United States are estimated to be diagnosed with sarcoma each year [2]. In Japan, the annual incidence of soft-tissue sarcomas is approximately 5500–6000 cases, with an incidence rate of around 3.22 per 100,000 people, however, bone sarcomas are much rarer, generally occurring at a rate < 1 per 100,000 people [3]. These tumors can develop across a wide age range. Soft-tissue sarcomas are more commonly observed in the elderly, particularly among people 60–70 years old, whereas bone sarcomas, such as osteosarcoma, are more prevalent among children and adolescents.

The mainstay of treatment for most patients with sarcoma is surgical resection, followed by limb or trunk reconstruction. Perioperative adjuvant chemotherapy and/or radiotherapy are administered according to the histological subtype. Despite the development of combined treatments, a significant proportion of patients with sarcoma respond poorly to chemotherapy, leading to local relapse or distant metastasis. The main cause of death associated with sarcoma is lung metastasis, which has an extremely poor prognosis. Therefore, tumor monitoring and early detection of recurrent or metastatic disease could improve patient prognosis. However, no reliable biomarkers meeting these demands are currently available. Typically, computed tomography, positron emission tomography, magnetic resonance imaging, scintigraphy, and X-ray are mostly employed to identify or track tumor growth. Only few studies have reported the usefulness of serological markers such as alkaline phosphatase (ALP) [4], lactic dehydrogenase (LDH) [5], and CA125 [6] in patients with osteosarcoma, Ewing sarcoma, and epithelioid sarcoma, respectively. Therefore, the development of novel circulating biomarkers to detect tumors or predict drug sensitivity is one of the most important challenges in sarcoma management.

### Liquid biopsy

Liquid biopsy is a revolutionary diagnostic technique based on the analysis of biomolecules such as circulating cells, nucleic acids, proteins, and metabolites contained in body fluids such as blood, urine, and saliva. The concept of liquid biopsy was first introduced for circulating tumor cells (CTCs) [7, 8] and rapidly extended to circulating tumor DNA (ctDNA) [9] and other tumor-derived products such as circulating cell-free RNA (noncoding and messenger RNA) [10], or extracellular vesicles [11]. Liquid biopsies have several advantages to tissue biopsies, including its noninvasive nature and potential for repeated testing in the same patient. In addition, liquid biopsy appears useful not only for diagnosis but also for predicting treatment response and prognosis, being expected to serve as basis for the development of the next generation of diagnostic and monitoring technology [12]. To date, accumulating evidence has shown that these techniques may serve as a novel tool for real-time monitoring or prognostic prediction for bone and soft-tissue sarcomas.

## Technologies used in liquid biopsy for bone and soft-tissue sarcomas

### Circulating tumor cells

CTCs are cancer cells released from a primary tumor or metastatic site into the bloodstream. These cells are critical in the metastatic cascade, potentially leading to the formation of secondary tumors in distant organs. First identified over 150 years ago, CTCs are valuable biomarkers for understanding tumor biology, predicting metastasis, and monitoring disease progression [13]. CTCs are rare in the bloodstream, often found at concentrations as low as one CTC per 10⁶–10⁸ leukocytes. Their rarity, combined with a high background of normal blood cells, poses significant challenges to their detection and isolation. Accordingly, highly sensitive and specific enrichment methods have been developed to address these hurdles [14].

**Fig. 1:**
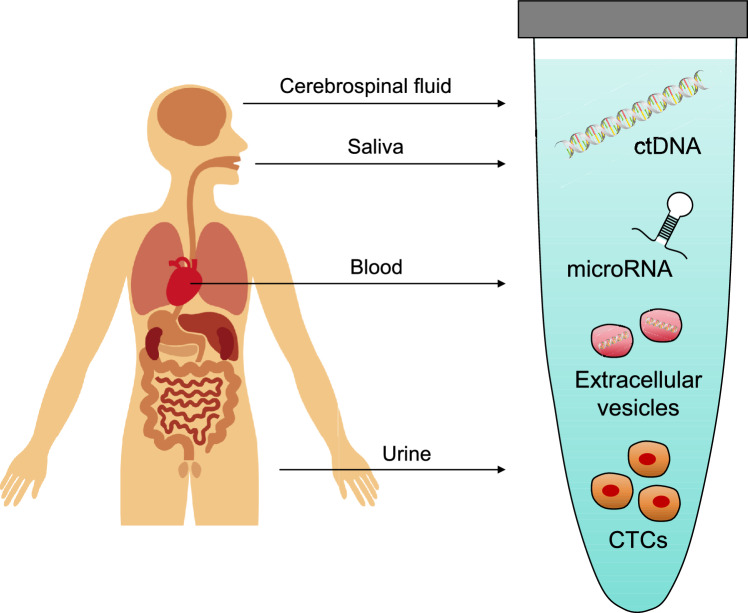
Circulating molecular targets used in liquid biopsy

CTC enrichment methods are developed based on the physical and biological properties of these cells [15, 16]. For example, size-based isolation techniques, such as the isolation by size of tumor cells method, separate CTCs based on their larger size compared with normal blood cells. Advanced systems like the MetaCell kit, which uses an 8 µm porous membrane, and CellSieve™ filters [17], which employ size-exclusion principles, have demonstrated efficacy in isolating CTCs from patients with various sarcoma subtypes, including osteosarcoma, chondrosarcoma, and rhabdomyosarcoma (Table 1). These antibody-independent options makes them particularly useful for capturing CTCs from sarcomas, where specific surface markers are not well characterized [18]. However, these methods are limited: white blood cell retention on microfiltration membranes can cause contamination, and smaller CTCs may escape capture, reducing the overall efficiency [19]. To overcome these limitations, innovative strategies to increase the CTC cluster size, such as selective size amplification using antibodies or binding agents, can improve capture rates and detection precision (Fig. 1).

**Table 1:** The CTC detection in patients with bone and soft-tissue sarcomas

Histological diagnosis	Molecular marker	Detection methods	Samples	Clinical/translational value				Reference	
				Diagnosis	Prognostic prediction	Tumor monitoring	Molecular analysis	Author	Year
Osteosarcoma	CK18^−^/CD45^−^	FISH	Whole blood	✔	✔			Zhang et al	2017
	Vimentin	FISH, whole-genome sequencing	Whole blood		✔			Satelli et al Li et al	2014 2017
	Type I collagen	RT-PCR	Whole blood	✔	✔			Wong et al	2000
	CSV	Immunofluorescence screening, ISH, whole-genome sequencing	Whole blood		✔	✔		Dao et al	2021
	GD2^2^CSV	Flow cytometry	Whole blood			✔		Fasanya et al	2021
	Hexokinase ^+^	Immunohistochemistry	Whole blood		✔			Mu et.al	2022
	BRIC5 (survivin)	CanPatrol™ CTC enrichment technology combined with in situ hybridization	Whole blood		✔			Lu et al	2023
	IMP3^+^	multiplex RNA in situ hybridization	Whole blood	✔				Dai et al	2023
Ewing sarcoma	Size-based microinfiltration	RT-PCR (*EWS-FLI-1/ERG*)	Whole blood		✔	✔		Hayashi et al	2017
	CD99	RT-PCR (*EWS-FLI-1/ERG*)	Whole blood		✔			Benini et al	2018
	β3-adrenorecepor	Flow cytometry	Whole blood		✔			Calvani et al	2020
Synovial sarcoma Leiomyosarcoma Liposarcoma	ISET	Immunocytochemistry (EGFR)	Whole blood			✔		Braun et al	2018
Soft-tissue tumors	ISET	Immunocytochemistry (Pan CK, CD45)	Whole blood				✔	Chinen et al	2014

Abbreviation: *FISH* fluorescence in situ hybridization, *ISET* isolation by size of tumor cells, *GD2* ganglioside 2, *CSV* cell surface vimentin, *IMP3* insulin-like growth factor mRNA-binding protein 3

Biological detection methods utilize tumor-associated surface proteins to identify CTCs. For example, the CellSearch system, the first FDA-approved technology for CTC detection, targets cells expressing the epithelial cell adhesion molecule (EpCAM) [20]. While effective for epithelial-origin cancers such as breast, colorectal, and prostate cancers, this method does not detect sarcomas, of mesenchymal origin. Alternative markers, such as cell surface vimentin, have been explored for sarcomas [21]. In addition, immunomagnetic separation techniques using markers such as CD99 for Ewing sarcoma [22] have demonstrated high viability in isolating functional CTCs.

RNA-based detection methods also provide options for distinguishing sarcoma cells from normal blood cells. These techniques rely on the unique gene expression profiles of sarcoma CTCs, such as *EWS-FLI1* fusion transcripts in Ewing sarcoma, to ensure accurate identification. The detection of *EWS-FLI1* fusion-positive CTCs has provided a valuable tool for early diagnosis, monitoring minimal residual disease (MRD), and predicting recurrence of Ewing sarcoma [23]. In osteosarcoma, CTCs expressing mesenchymal markers such as CSV or osteoblastic markers such as alkaline phosphatase [24] have been correlated with disease progression. In soft-tissue sarcomas, including leiomyosarcoma, synovial sarcoma, and liposarcoma, CTCs present unique challenges due to tumor heterogeneity [25]. Epidermal growth factor receptor (EGFR) positivity in CTCs has been observed in up to 93.75% of high-grade cases, suggesting the possibility of developing targeted therapies against EGFR [26]. In rhabdomyosarcoma, CTCs often display myogenic markers such as desmin and MyoD1, which may assist in their isolation and characterization.

Advances in CTC detection and enrichment technologies have enabled their capture and analysis, offering insights into tumor biology, metastasis, and therapeutic response. Despite current challenges such as heterogeneity among sarcoma subtypes and technical limitations in CTC isolation, ongoing research aims to refine these methodologies [27]. Although liquid biopsy targeting CTC has not yet achieved to clinics, integrating CTC analysis into routine clinical practice holds the potential to revolutionize sarcoma care.

### Circulating tumor DNA (ctDNA)

ctDNA comprises DNA fragments released into the bloodstream during tumor cell apoptosis, necrosis, or active secretion [28]. These fragments carry tumor-specific genetic and epigenetic alterations, providing noninvasive and dynamic insights into the tumor genome. Compared with traditional tissue biopsy, ctDNA analysis offers several advantages, including the ability to capture tumor heterogeneity, monitor real-time changes in tumor dynamics, and assess therapeutic efficacy.

Technologies for ctDNA detection include droplet digital PCR (ddPCR) [29] and next-generation sequencing (NGS) [30]. ddPCR provides high sensitivity and specificity, enabling detection of low-frequency mutations. This is particularly useful for monitoring specific mutations in sarcoma-related genes such as TP53 and RB1, commonly altered in bone and soft-tissue sarcomas (Table 2). NGS, conversely, allows comprehensive genomic profiling, including detection of multiple mutations, copy number variations, and structural rearrangements. This comprehensive approach is invaluable for identifying actionable targets and informing personalized treatment strategies.

**Table 2:** The ctDNA detection in patients with bone and soft-tissue sarcomas

Sarcoma type	Molecular target	Detection technique	Sample	Clinical implication			References	
				Diagnostic value	Prognosis	Monitoring of therapeutic effect	Author	Year
Osteosarcoma	TP53, ATRX, DLG2, MET mutations	tNGS	Plasma		✔		Barris et al	2018
	Chromosome 8q	ddPCR + NGS	Plasma		✔		Shulman et al	2018
	TP53, ATRX	NGS	Plasma			✔	Shah et al	2021
	4 CPGs (cg02169391,cg22082800,cg25680486,cg26100986)	ddPCR	Plasma		✔		Lyskjaer et al	2021
	Copy number abnormality	ULP-WGS	Plasma		✔		Audinot et al	2023
Ewing sarcoma	EWS/FLI, EWS/ERG	NGS, ddPCR	Plasma		✔	✔	Krumbholz et.al Hayashi et.al Schmidkonz et.al Krumbholz et al Anderson et al	2016 2016 2020 2021 2023
	STAG2, TP53	NGS	Plasma			✔	Shulman et.al Klega et.al	2018 2018
Chondrosarcoma	IDH1 and IDH2 mutations	ddPCR	Plasma		✔	✔	Gutteridge et al	2017
	IDH1/2 or GNAS mutation	ddPCR	Plasma	✔	✔		Lyskjær et al	2021
Dedifferentiated liposarcoma	MDM2	NGS	Plasma			✔	Przybyl et al	2022
Myxoid liposarcoma	t(12;16) and TERT C228T promoter mutation	qPCR	Plasma			✔	Braig et al	2019
Synovial sarcoma	SS18-SSX1 or SSX2 fusion sequence	tNGS	Plasma			✔	Eisenhardt et al	2022
Rhabdomyosarcoma	PAX3-FOXO1 fusion sequence	ddPCR	Plasma		✔	✔	Eguchi-Ishimae et al Tombolan et al	2019 2019
Leiomyosarcoma	TP53, RB1, PTEN	ULP-WGS	Plasma			✔	Hemming et al	2019
	CAPP-Seq, a genome-wide interrogation copy-number alterations	NGS	Plasma			✔	Przybyl et al	2018
	73-gene panel (including TP53, BRAF, CCNE, EGFR, PIK3CA)	tNGS	Plasma			✔	Arshad, et al	2020
Metastatic soft-tissue sarcoma	TP53, PIK3CA	tNGS	Plasma		✔		Eastley et al	2018

Abbreviation: *tNGS* targeted next generation sequencing, *ddPCR* droplet-digital polymerase chain reaction, *dPCR* digital polymerase chain reaction, *ULP-WGS* ultra-low passage whole-genome sequencing, *CAPP-Seq* cancer personalized profiling by deep sequencing

In the osteosarcoma biology study by the Children Oncology Group, an ultra-low-pass whole-genome sequencing assay in 72 patients with primary localized osteosarcoma detected ctDNA in 57% of newly diagnosed patients with osteosarcoma; further, the risk of events and death increased with ctDNA levels [31]. Another study using plasma samples from the prospective OS2006 trial used an ultra-low-pass whole-genome sequencing assay to detect copy number alteration in 183 patients. While the metastatic status at diagnosis is the main known prognostic factor in osteosarcoma, Audinot et al. reported that the copy number abnormality score at diagnosis (diagCPA) is a continuous variable independently associated with outcomes [32]. diagCPA was also a major prognostic factor at the time of surgery and until the end of treatment, independent of the histological response [32]. These data indicate that adding diagCPA to the metastatic status at diagnosis or poor histological response after surgery improves prognostic stratification.

Ewing sarcoma is characterized by the presence of *EWSR1* rearrangements with a member of the ETS family of genes [33]. The two most common fusion genes are *EWSR1-FLI1* and *EWSR1-ERG*, which occur in 85–90% and approximately 10% of cases, respectively [34, 35]. In the EWING2008 trial, genomic *EWSR1* fusion sequence spanning primers and probes were used for ctDNA quantification by digital droplet PCR in plasma samples from 102 patients with Ewing sarcoma. Accordingly, Krumbholz et al. reported that pretreatment ctDNA copy numbers correlated with event-free and overall survival. Interestingly, decreased ctDNA levels were observed in most cases after only two blocks of induction chemotherapy consisting of vincristine, ifosfamide, doxorubicin, and etoposide [36]. Recently, Sulman et al. demonstrated that a ctDNA burden ≥ 0.5% after one cycle of chemotherapy can identify patients highly likely to relapse, which may contribute to novel risk-adapted therapy trials focused on ctDNA burden [31].

ctDNA detected in other types of sarcoma include the *IDH1/2* mutation in chondrosarcoma [37], t(X;18)(p11;q11) in synovial sarcoma, which fuses *SS18* in chromosome 18 to *SSX1* or *SSX2* in chromosome X [38], and *PAX3-FOXO1* fusion in rhabdomyosarcoma [39]. In leiomyosarcoma, ctDNA analysis has uncovered frequent mutations in *ATRX* and *TP53*, associated with poor outcomes [40].

Despite its potential, ctDNA analysis faces several challenges in sarcoma. The low ctDNA concentration in the bloodstream, especially in sarcomas with low cellular turnover, requires the use of highly sensitive detection methods. Further, the heterogeneity of bone and soft-tissue sarcomas complicates ctDNA analysis, as a single mutation may not fully represent the molecular landscapes of the tumor. Multi-gene panels and whole-genome sequencing are emerging as potential solutions to better capture the complexity of sarcoma genomes.

### Circulating microRNA

microRNAs (miRNAs) are small noncoding RNA molecules with approximately 18–25 nucleotides that modulate the expression of multiple target genes, playing important roles in various physiological and pathological processes [41–44]. Reportedly, miRNAs are frequently upregulated or downregulated in various tumors, indicating that miRNAs act either as oncogenes or tumor suppressors [45, 46]. A previous report showed that tumor cells secrete miRNAs into the circulation [47]. Their high stability and tissue specificity arise from their encapsulation within extracellular vesicles, such as exosomes or microvesicles, or their association with lipoprotein complexes [45]. Since then, the analysis of circulating miRNA levels in serum or plasma has been considered a novel approach in liquid biopsy, with significant potential for diagnosis, monitoring, and prognosis of bone and soft-tissue sarcomas (Table 3).

**Table 3:** The ctRNA detection in patients with bone and soft-tissue sarcomas

Sarcoma type	Molecular target	Detection technique	sample	Clinical implication			References	
				Diagnostic value	Prognosis	Tumor monitoring	Author	Year
Osteosarcoma	miR-21	RT-qPCR	Serum		✔	✔	Yuan et al	2012
	miR-21, miR-143 (downregulated), miR-199a-3p (downregulated)	RT-qPCR	Plasma	✔	✔		Ouyang et al	2013
	miR-25-3p	RT-qPCR	Serum	✔	✔	✔	Fujiwara et al	2017
	miR-29 family (downregulated)	RT-qPCR	Serum	✔	✔		Li et al	2019
	miR-34b (downregulated)	RT-qPCR	Plasma	✔	✔		Tian et al	2014
	miR-101 (downregulated)	RT-qPCR	Serum	✔	✔		Yao et al	2018
	miR-133b (downregulated), miR-206 (downregulated)	RT-qPCR	Serum	✔	✔		Zhang et al	2014
	miR-124 (downregulated)	RT-qPCR	Serum	✔	✔		Cong et al	2018
	miR-148a	RT-qPCR	Serum	✔	✔		Ma et al	2014
	miR-191	RT-qPCR	Serum	✔	✔		Wang et al	2015
	miR-196a, miR-196b	RT-qPCR	Serum	✔	✔		Zhang et al	2014
	miR-421	RT-qPCR	Serum	✔	✔		Zhou et al	2016
	miR-497 (downregulated)	RT-qPCR	Serum	✔	✔		Pang et al	2016
	miR-542-3p (downregulated)	RT-qPCR	Serum	✔	✔		Li et al	2017
	miR-221	RT-qPCR	Serum	✔	✔		Yang et al	2015
	miR-491 (downregulated)	RT-qPCR	Serum	✔	✔		Wang et al	2017
	miR-22	RT-qPCR	Plasma	✔	✔	✔	Diao et al	2020
	miR-34a (downregulated)	RT-qPCR	Serum	✔	✔	✔	Lian et al	2022
	miR-375	RT-qPCR	Serum	✔	✔	✔	Liu et al	2018
	miR-194 (downregulated)	RT-qPCR	Serum	✔	✔	✔	Shi et al	2020
	miR-487a, miR-493-5p, miR-501-3p, miR-502-5p	RT-qPCR	Serum	✔			Huang et al	2019
	miR-133a	RT-qPCR	Serum	✔	✔		Liu et al	2022
	miR-429 (downregulated) miR-143-3p (downregulated)	RT-qPCR	Serum	✔	✔		Yang et al	2020
	miR-337-3p, miR-484, miR-582, miR-3677	RT-qPCR	Serum	✔	✔	✔	Luo et al	2021
	miR-221-5p	RT-qPCR	Serum		✔		Monterde-Cruz et al	2018
	miR-221	RT-qPCR	Serum		✔		Nakka et al	2017
	miR-140	RT-qPCR	Serum	✔			Green et al	2023
	miR-30a-5p (downregulated), miR-556–3p (downregulated), miR-200a-3p (downregulated), miR-582-5p (downregulated)	RT-qPCR	Serum			✔	Heidari et al	2024
	miR-663a	RT-qPCR	Plasma	✔			Huang et al	2019
	lncRNA UCA1	RT-qPCR	Serum	✔	✔		Wen et al	2017
	lncRNA TUG1	RT-qPCR	Serum	✔	✔		Ma et al	2015
Ewing sarcoma	miR-125b (downregulated)	RT-qPCR	Serum	✔			Nie et al	2015
	Panel of 62 miRNAs	RT-qPCR	Serum	✔			Crow et al	2022
Dedifferentiated liposarcoma	miR-3613-3p	RT-qPCR	Whole blood		✔		Frickle et al	2018
	miR-155	RT-qPCR	Plasma		✔		Feng et.al	2018
	miR-99a-5p, miR-146b-5p, miR-148b-3p, miR-195-5p, miR-223-3p, miR-500b-3p, miR-505-3p	RT-qPCR	Serum		✔	✔	Fricke et al	2015
Rhabdomyosarcoma	miR-26a (downregulated)	RT-qPCR	Plasma	✔	✔		Tombolan et al	2020
	miR-206 (downregulated)	RT-qPCR	Serum	✔			Miyachi et al	2010
Malignant peripheral nerve sheath tumor	miR-24, miR-214, miR-801	RT-qPCR	Serum	✔			Weng et al	2013

Abbreviation: *RT-qPCR* real time quantitative polymerase chain reaction

Numerous studies have been conducted on the circulating miRNA signatures in osteosarcoma [48]. For instance, miR-21, an oncogenic miRNA, is significantly overexpressed in blood samples of patients with osteosarcoma, being associated with a worse prognosis. Other miRNAs upregulated in patients’ blood include miR-25-3p, miR-29 family, miR-191, miR-196, miR-421, and miR-542-3p [49–54] (Table 3). Among these, miR-25-3p has an oncogenic function intracellularly and extracellularly, with a negative correlation between expression levels and the prognosis of patients with osteosarcoma [49, 55]. In contrast, miR-34a, which is downregulated in osteosarcoma tissues, is underexpressed in blood samples, and associated with advanced disease [52]. Similarly, circulating miR-143, miR-199a-3p, miR-101, miR-206, miR-124, and miR-497 are downregulated in patients with osteosarcoma [56–59] (Table 3). Circulating miR-320a levels are higher in the osteoblastic than in the chondroblastic subtype, whereas circulating miR-199a-3p levels were significantly low in the osteoblastic subtype [60]. In patients with Ewing sarcoma, serum miR-125b expression is decreased compared with that in healthy individuals [61]. Interestingly, patients with a poor response to chemotherapy showed a significant miR-125b downregulation (Table 3). 

In patients with dedifferentiated liposarcoma, miR-1246, -4532, -4454, -619-5p, and -6126 are highly expressed in human dedifferentiated liposarcoma cell lines, tissues, serum, and exosomes, and can be used as biomarkers for early diagnosis or treatment targets [62]. Rhabdomyosarcoma, the most common soft-tissue sarcoma in childhood, shows high expression levels of muscle-specific miRNAs (miR-1, miR-133a, miR-133b, and miR-206). In their analysis of muscle-specific miRNA levels in the blood serum of patients with RMS, Miyachi et al. found that normalized serum miR-206 exhibited the highest sensitivity and specificity among muscle-specific miRNAs [63]. Approximately 50% of MPNSTs, which typically originates from cells forming the nerve sheath such as Schwann and perineural cells, occur sporadically and the rest of them originate in patients with the autosomal dominant genetic disorder neurofibromatosis type 1 (NF-1). Weng et al. found higher miR-801 and miR-214 expression in the serum of sporadic MPNST patients and NF1 MPNST patients than in NF1 patients [64]. Moreover, miR-24 was significantly upregulated in NF1 MPNST patients. Therefore, combining the three miRNAs (miR-801, miR- 214, and miR-24) could serve to distinguish NF1 MPNST patients from NF1 patients [64].

Although circulating miRNA detection technologies face challenges related to sensitivity, specificity, and standardization, future research will focus on combining circulating miRNAs with other liquid biopsy markers such as ctDNA and extracellular vesicles to improve diagnostic accuracy [65]. Along with improvements in detection technologies, circulating miRNAs have a potential in the revolution of the management of bone and soft-tissue sarcomas, paving the way for personalized oncology.

### Circulating microvesicles

Extracellular vesicles (EVs) are small vesicles, 50 nm to 2 μm in size, released from the surface of several cell types into bodily fluids such as blood, saliva, milk, sweat, tears, and urine [66]. There are several classes of EVs, including exosomes, microvesicles, and apoptotic bodies, produced by different mechanisms. EVs play a critical role in intercellular communication by transferring biomolecules such as proteins, lipids, mRNA, and miRNA between cells. In the tumor microenvironment, EVs secreted by tumor cells can interact with surrounding cells, delivering biomolecules to influence processes like tumor growth, invasion, metastasis, and chemotherapy resistance [67]. EVs are small membranous vesicles composed of a lipid bilayer with a cystic structure and high molecular stability in body fluids. Recently, EVs have received considerable attention as a target of liquid biopsy. Since EVs express tetraspanin family proteins such as CD63, CD81, and CD9 [68, 69], circulating EVs could be detected by these proteins as well as other tumor-specific markers.

In their proteomic analysis of purified EVs from synovial sarcoma (SS) cell lines, Yokoo et al. identified 199 common proteins across EVs. Among them, monocarboxylate transporter 1 (MCT1) was identified as a surface marker of SS-derived EVs, highly expressed in SS patient-derived EVs compared with healthy individuals (Table 4) [70]. Most importantly, the serum levels of MCT1^+^ CD9^+^ EVs reflect the tumor burden in SS patients. Interestingly, positive MCT1 was observed in most SS specimens and its cytoplasmic/plasma membrane expression was significantly associated with worse overall survival, indicating the potential therapeutic target.

**Table 4:** The Detection of extracellular vesicles in patients with bone and soft-tissue sarcomas

Sarcoma type	Molecular target	Detection technique	Sample	Clinical implication			References	
				Diagnostic value	Prognosis	Monitoring of therapeutic effect	Author	Year
Osteosarcoma	TGFβ	Size-exclusion chromatography	Serum	✔			Baglio et al	2017
	EV-miR-101	qRT-PCR	Plasma		✔		Zhang et al	2020
	EV-PD-L1	Ultracentrifugation	Serum			✔	Yoshida et al	2024
Ewing sarcoma	UGT3A2	Ultracentrifugation	Plasma	✔			Turaga et al	2023
	ENO-1	Size exclusion chromatography, ultracentrifugation	Serum	✔		✔	Uotani et al	2024
Liposarcoma	EV-miR-25–3p, EV-miR-92a-3p	Size-based precipitation	Plasma	✔		✔	Casadei et al	2017
Synovial sarcoma	MCT1	Size exclusion chromatography, ultracentrifugation	Serum	✔		✔	Yokoo et al	2021

Abbreviations: *UGT3A2* UDP glycosyltransferase 3A2, *ENO-1* enolase-1, *MCT1* monocarboxylate transporter-1

Uotani et al. identified ENO-1 and CD99, a marker for the immunohistochemistry of ES, on EVs purified from the blood serum of patients with Ewing sarcoma before treatment (Table 4). In an animal model of Ewing sarcoma, ENO-1^+^ CD63^+^ EVs were elevated along with tumor growth and reduced after tumor resection. Importantly, increased ENO-1^+^ CD81^+^ EVs in the patient serum before treatments can distinguish patients with Ewing sarcoma from healthy individuals with an area under the curve of 0.92 and reflected the tumor burden in Ewing sarcoma patients during multidisciplinary treatments [71].

Since EVs contain genetic material such as mRNA, miRNA, or DNA [72], advances in high-throughput technologies such as exosomal RNA sequencing and proteomic profiling, will enable more comprehensive analyses of exosomal cargo, which may identify specific molecular markers for a various malignant diseases. Combining exosome analysis with other liquid biopsy targets, such as ctDNA and CTCs, could improve diagnostic accuracy and provide a comprehensive view of tumor dynamics. Of note, EV-based delivery systems are being explored for targeted therapies, leveraging their natural ability to transfer therapeutic agents directly to tumor cells [73].

## Conclusion and future directions

This review provides a comprehensive overview of the applications of liquid biopsy technology in bone and soft-tissue sarcoma. Among several technologies, ctDNA analysis in liquid biopsy has advanced to clinics in recent years. In colon cancer, the CIRCULATE-Japan GALAXY observational study demonstrated the prognostic value of ctDNA positivity during the MRD window with significantly inferior disease-free and overall survivals [74]. Furthermore, ctDNA positivity correlated with shorter overall survival in patients who experienced recurrence, indicating the utility of ctDNA monitoring for post-resection recurrence and mortality risk stratification that could guide adjuvant therapy [74]. Importantly, postsurgical ctDNA positivity identified patients who benefited from adjuvant chemotherapy [75]. Although liquid biopsy assays for sarcomas are still at an early phase, ctDNA detection may be promising for sarcoma subtypes with specific fusion genes. For sarcoma subtypes without specific genetic mutations, miRNA, EV, or CTC detection may be useful, together with the development of a quantification method. If these modalities could evaluate the presence of MRD, the findings would help physicians and patients decide whether adjuvant therapy should be administered or not. In patients who had undergone inadvertent excision at the previous hospital, observation would be suggested in patients with negative MRD; however, additional resection or adjuvant therapy would be indicated in patients with positive MRD. Ongoing evaluation of the efficacy of these modalities through larger, longitudinal trials is necessary to confirm the clinical significance of liquid biopsy in the management of bone and soft-tissue sarcomas.
